# Analysis of medication and prescription background risk factors contributing to oral medication administration errors by nurses: A case–control study

**DOI:** 10.1097/MD.0000000000030122

**Published:** 2022-08-19

**Authors:** Ryohei Suzuki, Takamasa Sakai, Mariyo Kato, Masaaki Takahashi, Akira Inukai, Fumiko Ohtsu

**Affiliations:** a Graduate School of Pharmacy, Meijo University, Nagoya, Japan; b Department of Pharmacy, National Hospital Organization Higashinagoya National Hospital, Nagoya, Japan; c Drug Informatics, Faculty of Pharmacy, Meijo University, Nagoya, Japan; d Department of Patient Safety, National Hospital Organization Higashinagoya National Hospital, Nagoya, Japan; e Department of Neurology, National Hospital Organization Higashinagoya National Hospital, Nagoya, Japan.

**Keywords:** administration error, case–control study, contributing factor, medication factor, prescription factor

## Abstract

Medication errors, including overdose and underdose, have a significant impact on patients and the medical economy. We need to prevent or avoid recurring medication errors. Therefore, we conducted a survey to identify medication and prescription background risk factors contributing to the administration of medication by nurses. This study surveyed cases of medication administration errors. This study was conducted at Higashinagoya National Hospital from April 1, 2018, to October 31, 2019. Patients’ backgrounds and medication and prescription background risk factors were investigated. Three control cases were randomly selected for each medication error case. We defined the group of medication error cases as the medication error group and the group of control cases as the no-medication-error group. A logistic regression analysis was performed for factors related to medication errors. A total of 202 patients were included in the medication error group. The median age and number of medications were 78 years and 7, respectively. A total of 606 cases were included in the no-medication-error group. The median age and number of medications were 77 years and 6, respectively. The factors that exhibited a relationship with the medication error group were the number of administrations per day, dosing frequency on indicated days, prescription and start dates were the same, medications from multiple prescriptions, and continuous use of a medication received prior to admission. This study identified existing medication and prescription background risk factors. Overlapping risk factors from these groups might contribute to medication administration errors. Therefore, reviewing these factors is necessary to avoid recurring medication administration errors.

## 1. Introduction

Medication errors are the most commonly reported errors in hospitals.^[1]^ The most frequently reported medication errors, including incorrect dose, incorrect medication, and omitted administration, occur during medication administration.^[2–5]^ Medication overdose, possibly due to administration of an incorrect dose, enhances the effect of the medication and sometimes leads to adverse effects, unnecessary hospitalization, and increased medical costs.^[6–8]^ Conversely, medication underdose, possibly due to omitted administration or administration of an incorrect dose, delays the patient’s treatment, deteriorates the condition, and prolongs hospital stay. Therefore, medication errors have a significant impact on patients and the medical economy; thus, it is necessary to avoid them and prevent their recurrence.

Medical professionals, mainly nurses, often manage oral medications in hospitals. This is because patients with deteriorated condition or decline in cognitive function are unable to self-manage medication. Furthermore, the administration of medication, such as narcotics, requires careful management. Common medications whose incorrect administration can result in medication errors include cardiovascular drugs, neurological drugs, anticoagulants, nonsteroidal anti-inflammatory drugs, and methotrexate.^[9–11]^ The association between the number of medications and medication errors has also been reported.^[6–9]^ Risk factors, such as identical prescription and treatment initiation dates, as well as medication that were prescribed by >1 physician and/or by the same physician at different times are important risk factors during medication administration errors in clinical practice. However, studies on the analysis of these risk factors have not been sufficiently conducted.

Clarifying the risk factors of medication errors is important for preventing the recurrence of medication administration errors, ensuring patient safety, and improving healthcare quality. Therefore, this study was conducted in an effort to identify the existing medication and prescription background risk factors related to medication administration errors.

## 2. Methods

### 2.1. Reporting system for medication administration error cases

At Higashinagoya National Hospital, all nurses who encountered or were related to a medication error promptly reported errors to their supervisor or departmental risk manager. They also wrote a medication error report.

We collected medication error reports of 2 types: for cases where patients were not subjected to incorrect actions by nurses, and for cases where patients were subjected to incorrect actions by nurses, regardless of their impact. The medication error reports included the date of occurrence, patient age, sex, specific details of the case, and countermeasures.

### 2.2. Surveyed cases

We surveyed cases of oral medication administration errors between April 1, 2018, and October 31, 2019. We excluded the same case reported in duplicate. The American Society of Health-System Pharmacists defines medication administration errors as omitted medication, incorrect dosage intervals, incorrect doses, incorrect medication, and/or incorrect patients.^[12]^ We excluded the survey cases of inadequate medication management such as dropped or broken drugs, cases caused by medication to be administrated as needed, cases caused by inadequate prescription orders or dispensing errors, and cases with insufficient information. Furthermore, incorrect patient errors were excluded from the survey cases because they were caused by factors other than the medication, such as the influence of the same name or lack of confirmation of the name of the patient. Then, we only analyzed oral medication administration error cases by nurses, including medication and prescription background factors.

### 2.3. Research items

This study retrospectively surveyed medication error factors such as patient background and medication, prescription background based on medication error reports, electronic medical records, and dispensing records such as prescriptions.

#### 2.3.1. Patient background.

This study surveyed the following parameters for patient background from medication error reports and electronic medical records: age, sex, primary medical department, modified Rankin Scale score, and route of administration.

#### 2.3.2. Medication and prescription background.

This study surveyed the following medication and prescription background factors from medication errors based on medication error reports, electronic medical records, and dispensing records: number of medications, number of administration per day, dosing frequency on indicated days, prescription and start dates were the same, orders prescribed by >1 physician and/or by the same physician at different times (medications from multiple prescriptions), continuous use of a medication received prior to admission, and not 1 package or 1 tablet at each dosage.

### 2.4. Research design and statistical methods

We conducted a case–control study to analyze the factors associated with medication administration errors. A patient who was confirmed to have a medication administration error was considered in the medication error case. The corresponding control cases were randomly selected from patients in the same ward on the same day that the medication administration error case was confirmed in the medication error cases. Additionally, we excluded self-administered cases, in which nurses did not manage medications for the corresponding control cases. Three control cases were randomly selected for each medication error case. The group with medication error cases was defined as the medication error group, and the group with control cases was defined as the no-medication-error group.

We compared medication error factors between the 2 groups using univariate logistic regression analysis. In addition, multivariate logistic regression analysis was performed by selecting patient background, medication and prescription background factors (*P* < .20 in univariate logistic regression analysis). The number of samples required for logistic regression analysis is >10 times the number of explanatory variables to be included in the prediction model.^[13]^ Then, we conducted a logistic regression analysis, provided that the sample size was at least 10 times the number of explanatory variables. Additionally, we calculated the correlation coefficients of each factor to check multicollinearity. Subsequently, multivariate logistic regression analysis was performed after confirming the absence of any correlation between each factor.

We also calculated medication and prescription background factors that showed a relationship in multivariate logistic analysis for medication error group and no-medication-error group. We compared the number of medication factors in the medication error group and no-medication-error group using the Mann–Whitney *U* test. The number of medications was a medication factor if the number of medications was 5 or more from the aspect of polypharmacy,^[14]^ and if the number of administrations per day was 3 or more from the aspect of adherence.^[15]^

The significance level was set at 5%. IBM SPSS Statistics Version 27 (IBM Corporation, Armonk, NY, USA) was used to analyze the data.

### 2.5. Ethical considerations

This study was approved by the Ethical Review Committee of the National Hospital Organization Higashinagoya National Hospital.

## 3. Results

### 3.1. Summary of medication administration error cases

**Figure 1.**
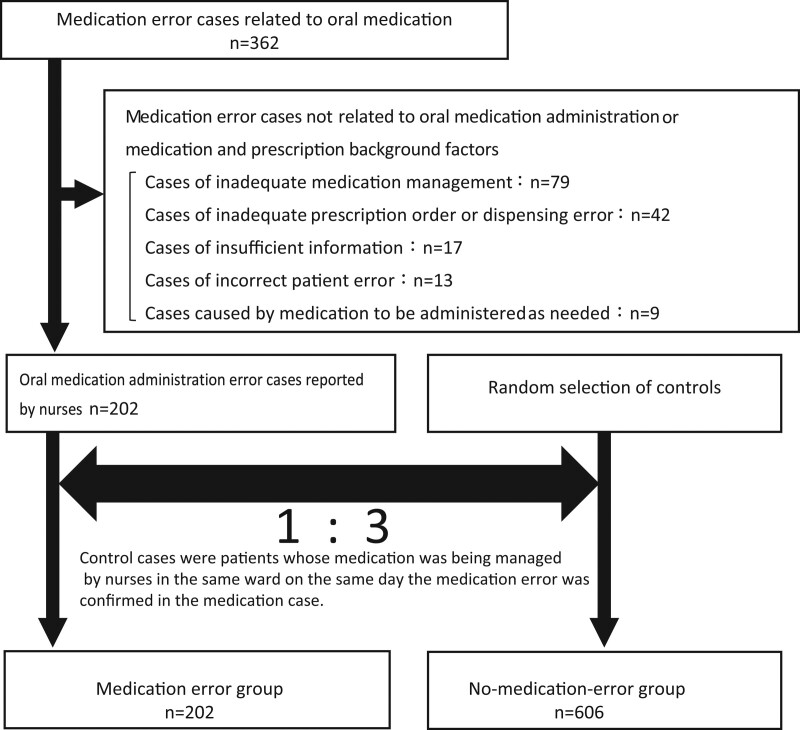
Summary of data extraction.

There were 362 cases of medication errors. This study excluded 79 cases of inadequate medication management, 9 cases caused by medication to be administered as needed, 42 cases of inadequate prescription order or dispensing error, 17 cases of insufficient information, and 13 cases of incorrect patient error; thus, a total of 202 medication administration error cases were analyzed in the medication error group. There were 606 cases in the no-medication-error group (Fig. 1).

### 3.2. Patient background, medication, and prescription background

Table 1 shows the patient background, medication, and prescription background factors for each group. The median age of the medication error group was 78 years (112 men and 90 women). The median age of the no-medication-error group was 77 years (282 men and 324 women). The primary medical departments included neurology and respiratory in both groups. The median number of medications was 7 in the medication error group and 6 in the no-medication-error group. The median number of administrations per day was 4 in the medication error group and 3 in the no-medication-error group.

**Table 1:** Patient background, medication and prescription background factors.

	Medication error group (n = 202)	No-medication-error group (n = 606)	*P* value
Age, median (IQR), yr	78 (67–83)	77 (67–84)	.9*
Sex, n (%)			
Men	112 (55)	282 (47)	.03†
Women	90 (45)	324 (53)	
Primary medical department, n (%)			
Neurology	99 (49)	288 (48)	.71†
Respiratory	29 (14)	91 (15)	.82†
Pediatrics	22 (11)	63 (10)	.84†
Neurosurgery	20 (10)	63 (10)	.84†
Orthopedic surgery	19 (9)	64 (11)	.64†
Others	13 (6)	37 (6)	.87†
mRS, n (%)			
0–3: Able to walk independently	52 (26)	92 (15)	<.01†
4–5: Unable to walk independently	150 (74)	514 (85)	
Route of administration, n (%)			
Oral	156 (77)	432 (71)	.10†
Not oral	46 (23)	174 (29)	
Number of medications, median (IQR)	7 (6–10)	6 (4–8)	<.01*
Number of administrations per day, median (IQR)	4 (3–5)	3 (3–4)	<.01*
Dosing frequency on indicated days, n (%)	8 (4)	5 (1)	<.01†
Prescription and start dates were the same, n (%)	38 (19)	34 (6)	<.01†
Medications from multiple prescriptions, n (%)	136 (67)	271 (45)	<.01†
Continuous use of a medication received prior to admission, n (%)	74 (37)	110 (18)	<.01†
Not 1 package or 1 table at each dosage, n (%)	189 (94)	525 (87)	<.01†

IQR = interquartile range, mRS = modified Rankin Scale.

* Mann–Whitney *U* test.

†χ ^2^ test.

The medication error factors that exhibited *P* < .2 in univariate logistic regression analysis were sex, modified Rankin Scale, route of administration, number of medications, number of administrations per day, dosing frequency on indicated days, prescription and start dates were the same, medication from multiple prescriptions, continuous use of a medication received prior to admission, and not 1 package or 1 tablet at each dosage.

Table 2 shows the results of multivariate logistic regression analysis. Medication error factors that exhibited a relationship with the medication error group were the number of dose administrations per day, dosing frequency on indicated days, prescription and start dates were the same, medications from multiple prescriptions, and continuous use of a medication received prior to admission. The medication error group in this study included 202 cases, which was 10 times more than the explanatory variables in the multivariate logistic analysis; thus, the required sample size was satisfied. Moreover, the correlation coefficients were <0.6 for each factor, and multicollinearity was not observed.

**Table 2:** Results of multivariate logistic regression analysis of patient background, medication and prescription background factors.

	Medication error group (n = 202)	No-medication-error group (n = 606)	Odds ratio	95% CI	*P* value
Sex, n (%)					
Men	112 (55)	282 (47)	1.4	1.0–2.0	.06
mRS, n (%)					
0–3	52 (26)	92 (15)	1.4	0.9–2.2	.13
Route of administration, n (%)					
Oral	156 (77)	432 (71)	0.9	0.6–1.4	.74
Number of medications, median (IQR)	7 (6–10)	6 (4–8)	1.1	1.0–1.1	.11
Number of administrations per day, median (IQR)	4 (3–5)	3 (3–4)	1.2	1.1–1.4	<.01
Dosing frequency on indicated days, n (%)	8 (4)	5 (1)	6.3	1.9–21.0	<.01
Prescription and start dates were the same, n (%)	38 (19)	34 (6)	2.8	1.7–4.8	<.01
Medications from multiple prescriptions, n (%)	136 (67)	271 (45)	1.7	1.2–2.4	<.01
Continuous use of a medication received prior to admission, n (%)	74 (37)	110 (18)	2.0	1.3–3.0	<.01
Not 1 package or 1 table at each dosage, n (%)	189 (94)	525 (87)	1.0	0.5–2.0	.97

CI = confidence interval, IQR = interquartile range, mRS = modified Rankin Scale.

### 3.3. Number of medication and prescription background factors that showed a relationship with medication administration error

The medication and prescription background factors that showed a relationship with medication errors were number of administrations per day, dosing frequency on indicated days, prescription and start dates were the same, medications from multiple prescriptions, and continuous use of a medication received prior to administration. The median number of medication error factors that showed a relationship with the medication error group was 2, and that with the no-medication-error group was 1 and was significantly higher in the medication error group (Table 3).

**Table 3:** Number of medication and prescription background factors that showed a relationship with medication administration errors.

	Medication error group	No-medication-error group	*P* value
Number of medication and prescription background factors, median (IQR)	2 (1–3)	1 (1–2)	<.01*

IQR = interquartile range.

* Mann–Whitney *U* test.

## 4. Discussion

This study identified medication and prescription background risk factors in medication administration errors. It was found that risk factors, such as the number of administrations per day, dosing frequency on indicated days, prescription and start dates were the same, medications from multiple prescriptions, continuous use of a medication received prior to admission showed a relationship with medication administration errors.

The number of administrations per day showed a relationship with medication administration errors. There are multiple parameters that need attention during medication administration, such as understanding the medication instructions, confirming the medication route, dose, and medication name, and administering the medication.^[3,16,17]^ When the number of administrations per day became high, the chances for omissions and mistakes by nurses in each check also increased. This may have been a factor in medication administration errors.

The presence of prescription and start dates were the same showed a relationship with medication administration errors. It is necessary to add or review medication when there are changes in the patient’s condition and when the prescription is suddenly changed. Therefore, insufficient communication between physicians and nurses, and among nurses may have had an effect. It has been empirically understood that prescription date and start date were the same factors in medication administration errors. However, actual data are not shown. This study was able to clarify that prescription and start dates were the same factors in medication error. Medications with prescription and start dates were same and must be administered promptly to patients. This is because they are urgent medications for the patient’s condition. Therefore, it is difficult to avoid medications with prescription and start dates were the same in actual clinical practice. The importance of communication between medical professionals has also been suggested to avoid the occurrence of medication errors.^[18]^ Therefore, it is necessary to accurately communicate information and confirm the latest prescription details of patients, especially when the medication prescription and start dates were the same.

The presence of medications from multiple prescriptions showed a relationship with medication administration errors. The reason for this is thought to be the addition or change of medications owing to diagnosis by multiple department physicians or changes in medical conditions. This may result in multiple prescription configurations at each time of administration, making drug management more complex. This also supports the WHO’s Patient Safety Curriculum Guide for Medical Schools and points out that multiple prescriptions from different physicians should be considered as a background factor for patients and should be considered when a medication error occurs.^[19]^

Moreover, the dosing frequency on indicated days of medication showed a relationship with medication administration errors. Administering daily medications is an integral part of daily practice. However, irregularly administered medications, such as medications administered on alternate days or less frequently, are different from routine daily doses. As a result, nurses tend to omit tasks such as conformation, thus leading to medication errors. Therefore, it is necessary to share information on patients’ latest prescriptions and dose timings to prevent medication errors.

It has been reported that the number of medications is a factor in medication errors.^[6,9]^ However, the results of this study did not show a relationship with the number of medications used. Previous studies have focused on the number of medications.^[6,9]^ However, the prescription context, such as medications from multiple prescriptions or prescription and start dates were the same, has not been sufficiently examined. In view of this, it is necessary to confirm not only the number of medications but also the medication factors, such as the prescription background, when the number of medications is high. These medications need to be reduced.

The number of medication and prescription background factors was significantly higher in the medication error group than in the no-medication-error group. The Medication Regimen Complexity Index (MRCI) has been reported as an index of prescription complexity.^[20]^ The number of medications and dosing frequency on the indicated days were included in the MRCI. However, considering the complexity of the MRCI scoring process and the fact that it does not include medication factors, such as prescription and start dates were the same, medications from multiple prescriptions, and continuous use of a medication received prior to admission. Conversely, the number of medication and prescription background factors includes factors that have a significant impact on the occurrence of medication administration errors. Therefore, it can be easily verified that in clinical settings, it may be easily used to predict medication administration errors. And if there were many medication and prescription background factors, we need to reevaluate the current drug therapy based on the patients’ condition and living environment and consider reducing the number of medication factors. So, it can be expected to reduce medication errors and prevent the recurrence.

As some factors such as the timing and method of prescribing are controllable by medical professionals, it is possible to intervene by reviewing medication error factors in clinical practice. The limitations of this study are as follows: The data used for the study were a collection of spontaneous reports by medical professionals, and it is possible that some medication errors were overlooked or unnoticed. Additionally, the data used for this study were obtained from only 1 hospital that focused on patients with chronic diseases with long hospital stays. This may not represent all hospitalized patients.

## 5. Conclusions

This study identified the medication and prescription background risk factors contributing to medication administration errors: the number of administrations per day, dosing frequency on indicated days, prescription and start dates were the same, medications from multiple prescriptions and continuous use of a medication received prior to admission. A few studies have analyzed medication administration errors from the viewpoint of medication and prescription background factors. The results of this study are expected to contribute to the prevention of medication administration errors.

## Acknowledgments

We thank the participants of this study for their time and thoughtful contributions as well as Sanae Niwa (Department of Nursing) and Yuko Inoue (Department of Nursing) for their comments on earlier drafts of this manuscript.

## Author contributions

**Conceptualization:** Ryohei Suzuki, Takamasa Sakai, Fumiko Ohtsu.

**Data curation:** Ryohei Suzuki, Mariyo Kato, Masaaki Takahashi.

**Formal analysis:** Ryohei Suzuki, Takamasa Sakai, Fumiko Ohtsu.

**Investigation:** Ryohei Suzuki, Mariyo Kato, Masaaki Takahashi.

**Methodology:** Ryohei Suzuki, Takamasa Sakai, Akira Inukai, Fumiko Ohtsu.

**Project administration:** Ryohei Suzuki, Masaaki Takahashi, Akira Inukai, Fumiko Ohtsu.

**Supervision:** Masaaki Takahashi, Akira Inukai, Fumiko Ohtsu.

**Visualization:** Suzuki Ryohei, Takamasa Sakai, Mariyo Kato, Masaaki Takahashi.

**Writing – original draft:** Suzuki Ryohei, Takamasa Sakai, Mariyo Kato, Fumiko Ohtsu.

**Writing – review & editing:** Masaaki Takahashi, Akira Inukai, Fumiko Ohtsu.
